# Regenerative collagen biomembrane: Interim results of a Phase I veterinary clinical trial for skin repair

**DOI:** 10.12688/f1000research.15138.1

**Published:** 2018-06-12

**Authors:** Andreas Kaasi, João F. Lima-Neto, José A. Matiello-Filho, Mário H.S. Calejo, André L. Jardini, Paulo Kharmandayan

**Affiliations:** 1 Eva Scientific Ltd, São Paulo, 047040-030, Brazil; 2 National Institute of Biofabrication, Campinas, 13083-852, Brazil; 3 University of Campinas, Cidade Universitária , Campinas, 13083-970, Brazil; 4 Sorocaba Veterinary Hospital, Sorocaba, 18046-700, Brazil

**Keywords:** biomembrane, biofabrication, tissue engineering, medical device, collagen, wound healing

## Abstract

**Background:** The availability of commercial tissue engineering skin repair products for veterinary use is scarce or non-existent. To assess features of novel veterinary tissue engineered medical devices, it is therefore reasonable to compare with currently available human devices. During the development and regulatory approval phases, human medical devices that may have been identified as comparable to a novel veterinary device, may serve as predicate devices and accelerate approval in the veterinary domain. The purpose of the study was to evaluate safety and efficacy of the biomembrane for use in skin repair indications.

**Methods:** In the study as a whole (3 year total length), 15 patients (animals), dogs and cats (male/female, <8 years) with skin lesions of different etiologies considered difficult to heal (size, >2 cm), with a wound depth equivalent to 2nd/3rd degree burns are to be studied from Day 0 to Day 120-240, post-application of the biomembrane. This interim report covers the 5 patients assessed to date and deemed eligible, of which 3 enrolled, and 2 have completed the treatment. Wound beds were prepared and acellular collagen biomembranes (Eva Scientific Ltd, São Paulo, Brazil) applied directly onto the wounds, and sutured at the margins to the patient's adjacent tissue. Wound size over time, healing rate, general skin quality and suppleness were assessed as outcomes. Qualitative (appearance and palpation) and quantitative (based on Image Analysis of photographs) wound assessment techniques were used.

**Results:** Both patients’ wounds healed fully, with no adverse effects, and the healing rate was comparable in both, maxing out at approximately 1 cm ^2^/day.

**Conclusions:** Early results on the biomembrane's safety and efficacy indicate suitability for skin repair usage in veterinary patients.

## Introduction

Ever since the pioneering work of Rheinwald & Green (1975), on skin epidermis (keratinocyte) cultures, Yannas & Burke (1980) on hybrid (biologic/synthetic) dermis/epidermis skin substitutes, and Bell *et al*. (1981) on fully biologic dermis and epidermis skin substitutes, a number of companies and products engaged with tissue engineering technologies have developed and consolidated in the market. In particular, early clinical research (Phase 1 and Early Phase 1) has tended to be characterized by a tight relationship between the industry-academia-hospital stakeholders of this archetypical medical device triad.

The tissue engineered devices that have made the most headway, clinically and commercially, include Epicel®, Integra®, Dermagraft®, Apligraf® and Alloderm®. These products have all had to make decisions and compromises whether to mimic only the dermis, only the epidermis, or both. Whether to employ only synthetic biomaterials, only natural biomaterials, or both. And whether to include only living dermis-type (fibroblast) cells, only epidermis-type (keratinocyte) cells, or both, or alternatively, to let the device be acellular. Review articles written by Eisenbud *et al.* (2004) and Groeber *et al.* (2011) provide a more in-depth comparison of these products and others.

Tissue engineered devices for veterinary use are non-existent, or scarce, at best. The same can be said about veterinary clinical trials in general, which lack a global consensus concerning the rules and regulations governing them. Usually, veterinary clinical trials with *patients having spontaneously occurring diseases* are treated analogously to animal experimentation with *experimental subjects with induced diseases* ( Fürdös *et al*., 2015), requiring Ethics Committee approval for the use of Animals for Experimentation (the same way that this is required for “traditional” animal experimentation). By contrast, countries may not regard animal clinical trials as animal experiments, thus not requiring an Ethics Committee approval by law. This would present a *less* restrictive approach. A third possibility would be to implement a set of rules and regulations specific for veterinary clinical trials, with a separate Ethics Committee for this purpose. This would present a *more* restrictive approach.

Many researchers debating on the topic of veterinary clinical trials ( Fürdös *et al*., 2015; Vail, 2007) acknowledge the attractiveness of letting veterinary clinical trials pave the way for human use of the same therapy. To make the cross-applicability of veterinary/human data as smooth as possible, and while more specific veterinary clinical trial rules and regulations remain absent or non-enforced, it may be advantageous to frame veterinary clinical trials to conform with rules, regulations and standards governing human clinical trials. One aspect of interest is the sample size and terminology of the “phase” of the trial.

In the human domain, the U.S. Food and Drug Administration (FDA) defines the number of participants appropriate for each phase as follows: Phase 1, 20–100 participants; Phase 2, “several hundred” participants; Phase 3, 300–3000 participants; Phase 4, “several thousand” participants ( https://www.fda.gov/ForPatients/Approvals/Drugs/ucm405622.htm, retrieved on 12 Jan 2018).

The U.S. National Institutes of Health (NIH) has a similar definition for Phases 1 (n= 20–80) and 3 (n=100–3000), but offers a precise definition for Phase 2, at 100–1000 participants. The NIH, unlike the FDA, does not provide any specific participant number for Phase 4 ( https://www.nih.gov/health-information/nih-clinical-research-trials-you/basics, retrieved on 12 Jan 2018).

A so-called “Early Phase 1”, previously known as Phase 0, is defined as an “exploratory study involving very limited human exposure to the drug, with no therapeutic or diagnostic goals” ( https://clinicaltrials.gov/ct2/help/glossary/phase, retrieved on 12 Jan 2018). Again, the descriptor “very limited human exposure” does not offer precise guidance on numbers, but a figure of <15 participants appears to be the consensus for this type of study ( Gawai *et al*., 2017).

We believe a sample size of 15, being the borderline number between the above mentioned “Early Phase 1” and “Phase 1” (as defined by FDA and NIH, above) is a suitable sample size for veterinary clinical trials, being an economically and practically feasible number to execute in a veterinary medicine context (usually not backed up by heavy industry or institutional funding), whilst at the same time offering the desired property of translationability of veterinary clinical trial findings to human applications.

In this study, we set out to treat skin lesions in dog and cat patients referred to our clinical practice, where conventional clinical treatment had proved itself incapable of satisfactorily healing the lesions. In order to form suitable exclusion criteria with respect to wound size, we considered the following reference parameter: maximum lesion/defect size for a spontaneous and unconfined tissue regeneration to occur *in vivo*, without special treatment = 1 cm ( Dorin *et al*., 2008). The same value is also given in the literature in a burn context, when faced with a full thickness, third degree burn ( Papini, 2004). Thus, we chose to concentrate on wounds larger than this reference size.

In assessing the wounds and progression of healing, for qualitative data, we ordered that photographs be taken as often as possible, and, inasmuch possible, using certain reference angles of photography that were easy for the patient owners to employ when sending photographs from home. These same photographs formed the basis for a quantitative wound healing assessment, using the photographs as raw data for an image analysis powered calculation of wound area/healed area over time. The approach chosen required minimal or no investment, as opposed to commercial wound image scanner devices (see e.g., Bills *et al*., 2016; Davis *et al*., 2013) that can cost upwards of US$10,000. In addition to the experiences sought through this clinical trial, pertaining to the clinical outcome of the study, the experiences obtained with this image analysis method were seen as secondary benefits of the study that also would be useful to perfect the methodological aspects of the study for the remainder of the study period.

## Methods

This study has been reported using CONSORT guidelines (see Supplementary File 1 for a completed checklist).

### Study design

The protocol to this ongoing, 36-month, phase I, quasi-experimental, interventional clinical trial is available as Supplementary File 2. ^1^

**Figure 1.**
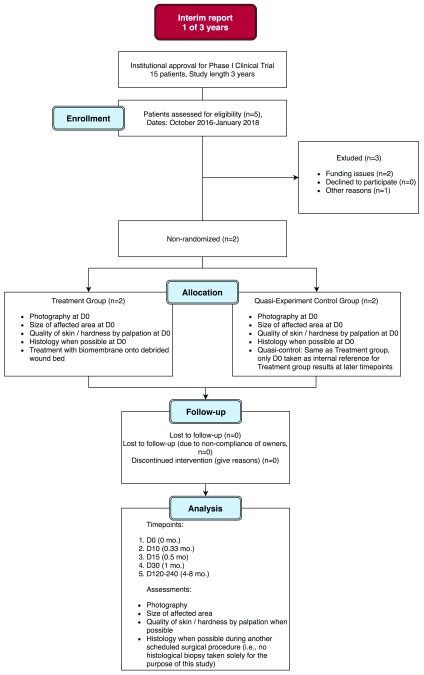
Study flow diagram.

Results in this interim report include data from animals who were assessed and eligible (n=5) and enrolled (n=3) in the trial, with treatment completed (n=2), at the time of writing ( Figure 1). Therefore this article presents interim results, spanning 1 year of the 3 year study length.

The study protocol abided by the principles of the WMA Declaration Of Helsinki – Ethical Principles For Medical Research Involving Human Subjects ( https://www.wma.net/policies-post/wma-declaration-of-helsinki-ethical-principles-for-medical-research-involving-human-subjects/), adapted to veterinary practice.

The Ethics Committee of Sorocaba Veterinary Hospital approved the study protocol before the study began (Ref. no. 20161012-1), and all patient (animal) owners provided written informed consent for their pets’ participation before undergoing any procedure related to the present study. The study was carried out at Sorocaba Veterinary Hospital.

### Study objective

The objective of the present study was to obtain clinical experiences with the use of a proprietary collagen biomembrane medical device for skin repair in dogs and cats, and to assess its safety and efficacy for this indication.

### Eligibility criteria

Dogs and cats, of both sexes, aged up to 8 years, with an etiology of skin lesion of different causes, such as burns, traumas, surgical excisions, were deemed eligible for treatment with the collagen biomembrane. The lesions should be equivalent to second or third degree burns, i.e., extending into the dermis or subcutaneous layers of the skin, or loss of skin tissue altogether. The size of the lesion should measure 2 cm or more (length/width/diameter as per the lesion geometry). This figure is double the reference maximum lesion/defect size (1 cm) for spontaneous and unconfined tissue regeneration *in vivo* to occur, without special treatment, as noted above. The rationale of setting the size criterion at *double* that figure, considered the maximum size for spontaneous healing (1 cm), was that the healing of a lesion/defect size of this magnitude (2 cm) would be unquestionably attributed to the treatment regime. Key exclusion criteria included lesions only of the epidermis (equivalent to a first degree burn), in addition to skin lesions stemming from chronic disease. These criteria are summarized in Table 1.

**Table 1.** Eligibility criteria for the present study.

Inclusion criteria	Exclusion criteria
Species: dog or cat Sex: male or female Age: <8 years Skin lesion etiology: various Skin lesion severity: equivalent to 2nd or 3rd degree burn Skin lesion size: > 2 cm (L/W/diameter as per geometry)	Epidermis-only lesions (equiv. 1st degree burn) Skin lesions stemming from chronic disease

### Surgical regimen

Each patient was photographed on the operating table, just prior to the first surgical procedures, from a variety of angles. Inasmuch possible, a decision on the most suitable angle and focal distance for future photographs, would take place during these initial photographs. Ideally, a single angle and focal distance would provide for a general wound healing assessment, as supposed to requiring multiple angles, especially since later follow-up time points depend on owners’ own photographs, with little or no control by study researchers. A ruler would be placed adjacent to the treatment area for later dimensional analyses, or, alternatively, direct measurements would be taken of reference distances such as eye-midline, knee-hip joint, and recorded on file.

Under general anesthesia (preanesthetic agents: acepromazine 0.02 mg/kg and meperidine 3 mg/kg, both administered intramuscularly; induction agent: propofol, 3 mg/kg, administered intravenously; maintenance agent in cats: propofol, 0.2 mg/kg/min; maintenance agent in dogs: oral isoflurane administered through tracheal intubation with a universal anesthetic vaporizer, dose being subject to anesthetist’s clinical assessment and varied as required during the surgery), the treatment area was prepared by shaving away fur adjacent to the treatment area and cleaned with saline and iodine. If the affected area had been subject to natural scarring, with the formation of hard, unhealthy, fibrotic scar tissue, this was surgically removed, exposing subcutaneous tissue. In this case, the tissue would be fixed in 10% formalin and submitted to histological analysis, using standard procedures. If the treatment area was an unhealed, deepithelialized wound bed, but with no fibrotic scar tissue formation, then shallow scalpel incisions were made scattered throughout the treatment area, causing local bleeding, with the aim of promoting regeneration and integration with the later application of the collagen biomembranes. In any case, debridement of lacerated, devitalized or contaminated tissue was performed prior to the application of the collagen biomembranes.

**Figure 2.**
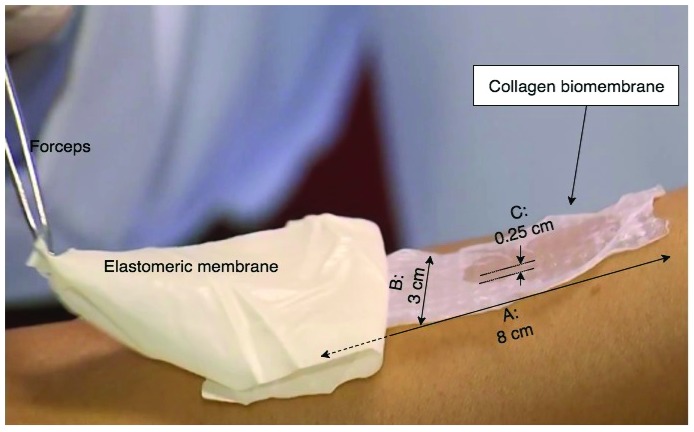
Regenerative collagen biomembrane (Eva Scientific Ltd, São Paulo, Brazil). The biomembrane measures 8×3 cm, and is scattered with small ridges throughout the structure, measuring approximately 100 μm in height, and a spacing of 0.25 cm between each elevated ridge. The standard presentation of the biomembrane is with a synthetic elastomeric membrane underlying the biomembrane itself; when applied to the treatment area, the device (double-layer synthetic membrane / biomembrane structure) is placed biomembrane-side down onto the treatment area, and the synthetic membrane carefully peeled away with a forceps, leaving behind the biomembrane (see Supplementary File 3 for footage of this application technique).

Acellular collagen biomembranes measuring 8 × 3 cm were obtained from Eva Scientific Ltd, São Paulo, SP, Brazil ( http://www.evascientific.com/products/biomembranespet/). According to the manufacturer, the biomembranes are made aseptically from collagen type I sourced from rat tail tendon (Ref. E-003RT050M1), using the manufacturer’s proprietary biofabrication apparatus. The resulting biomembranes possess a mean thickness of 200 to 300 μm, with a regular pattern of local depressions/elevations of approximately 100 μm and 2.5 mm apart center-to-center (see Figure 2).

Depending on the size and shape of the treatment area, one or several collagen biomembranes were used to cover the area, extending over the wound margin by a few millimeters. At the margins, the biomembranes were sutured to non-treatment tissue using the simple interrupted suture technique. If one or more biomembrane edges were facing away from the non-treatment tissue margin (i.e., into the wound, kissing or overlapping another biomembrane’s non-treatment tissue margin), the surgeon would make a decision on whether or not to make an internal suture; the biomembrane’s capacity to adhere to the wound bed and commence integrative regeneration discourages the use of the internal suture.

After applying and suturing all biomembranes, alginate hydrogel (Curatec ^®^, LM Farma, São José dos Campos, SP, Brazil) was generously applied onto the treated areas. At this stage, the patients’ general anesthesia would be retracted. The patients were then medicated with a long-duration injectable antibiotic drug (cefovecin sodium, 8 mg/kg), and the treatment areas covered up with gauze tape.

### Wound treatment regimen

Upon awakening from the anesthesia, the first dose of an antifungal oral drug (ketoconazole, 10 mg/kg or itraconazole 5 mg/kg) was administered, with the treatment carried through for the duration as prescribed by the drug manufacturer.

The sutures were removed upon attesting that the biomembrane had adhered well to adjacent tissue structures and when the sutures would not provide any useful additional mechanical support to the host tissue-wound-biomembrane system integrity. This could happen as early as Day (D)3 after the surgery.

Approximately every two days, the gauze tape was removed, and the wound cleaned with physiological saline and extra alginate hydrogel applied, as needed, and covered up again with fresh gauze tape, to prevent infection. This care was continued until the wound had completely healed.

### Patient evaluation and follow-up

Follow-up time points for patient evaluation were defined as D0 post-surgery, D10, D15, D30 and a long-term time point at D120–D240 (4–8 months). At these time points, patients had photographs taken of the treatment area at roughly identical angles and distance to those taken immediately prior to the surgery. These photographs were used as basis for quantitative size assessments, as detailed below. When possible, a qualitative assessment of skin tissue quality would be done, either by a researcher associated with the study or by the owner. If another surgical procedure was scheduled for a study participant, for any reason, a small skin biopsy was attempted to be taken from the treatment area, but the study’s premise was that end-point histology would not be necessary to provide assertive conclusions to the treatment effectiveness, based on clinical assessment and healing rates.

The wounds were monitored for adverse effects such as infection, stagnation of healing for more than 15 days, and necrosis. If any adverse effects were confirmed, in the first 1–2 days, then the biomembranes were excised or scraped off host tissue. If adverse effects were confirmed in later stages (>D3), the biomembranes were not excised due to the fact that by this time they had absorbed significantly into the host tissue, rendering removal difficult or impossible. In this case, the patients were treated symptomatically and added to the “Discontinued intervention” group, as indicated in the Study Flow Diagram ( Figure 1).

### Quantitative wound size assessments based on Image Analysis

In order to use a quantitative wound size measurement method based on two-dimensional image analysis, the photographs would ideally depict the wounds at an angle as normal (90°) as possible to the idealized, hypothetically two-dimensional wound area.

**Figure 3.**
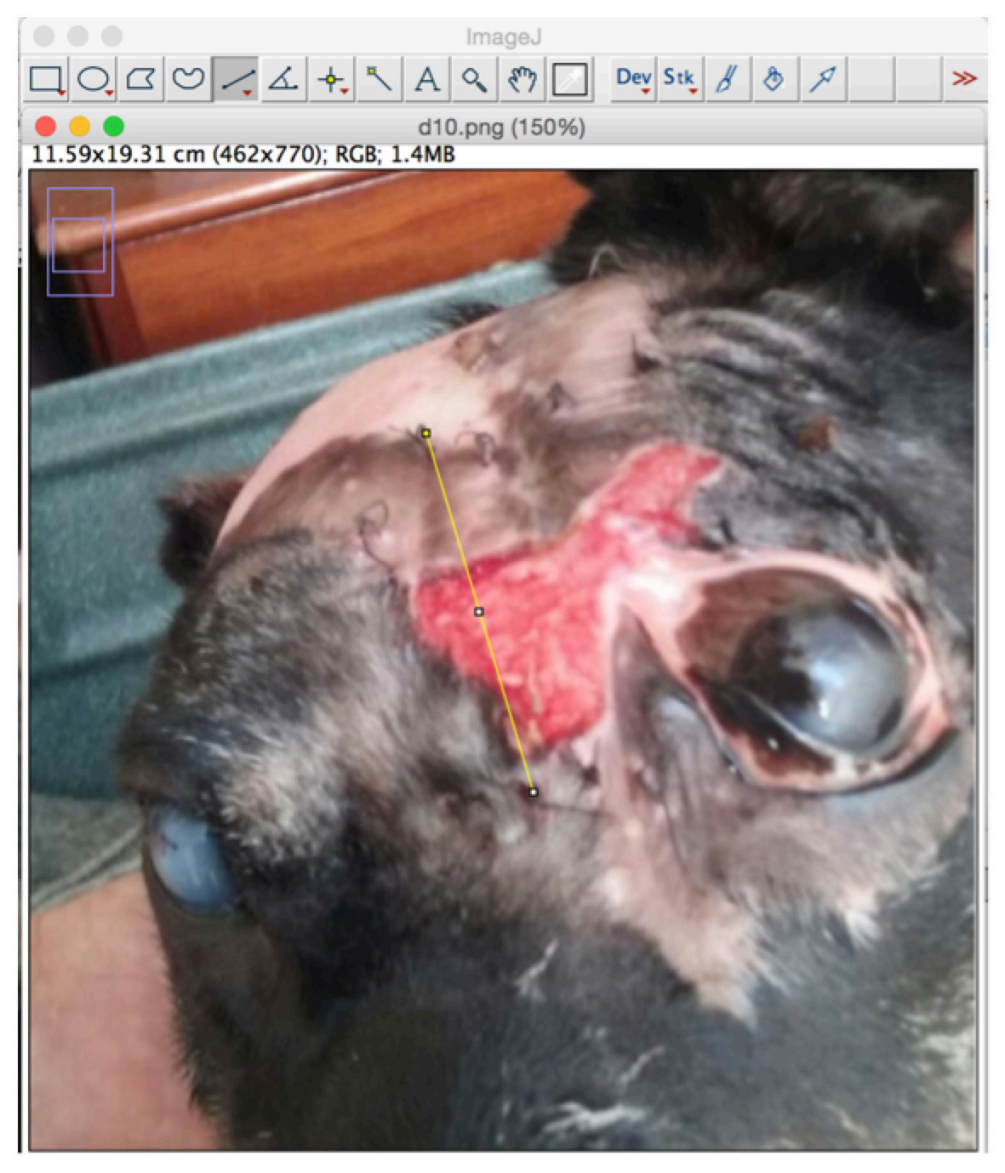
ImageJ software image analysis approach for quantitative size measurements of wounds - Part 1. The first part of the method is to use the Set scale function on the image analyzed for a known distance, visible on the photo. In this case, the distance chosen was the length of the biomembrane (with the sutures used for cue points of approximate termini of the biomembrane, useful for later time points when the original biomembrane had been completely absorbed into the patient tissue), with a correction applied to account for the inclination of the target plane, using a trigonometric approach.

Using ImageJ 1.49v ( Schneider *et al*., 2012) and the “Straight line” tool, a straight-line was traced for a known distance. Usually, the tool was applied to trace the long dimension of a biomembrane, using sutures applied at either end as cue points for the extremities, and, assuming the sutures were placed some 2 millimeters from the edges of the biomembranes, using the “Set scale” tool that distance in *pixels* (as traced in the previous step) was taken to be equivalent to a distance in *centimeters*, 7.5 cm, slightly less than the nominal 8.0 cm biomembrane length ( Figure 3). Subsequent uses of ImageJ’s “Measure” tool then output a distance in cm (distance) or cm ^2^ (area), which were used for the analyses.

**Figure 4.**
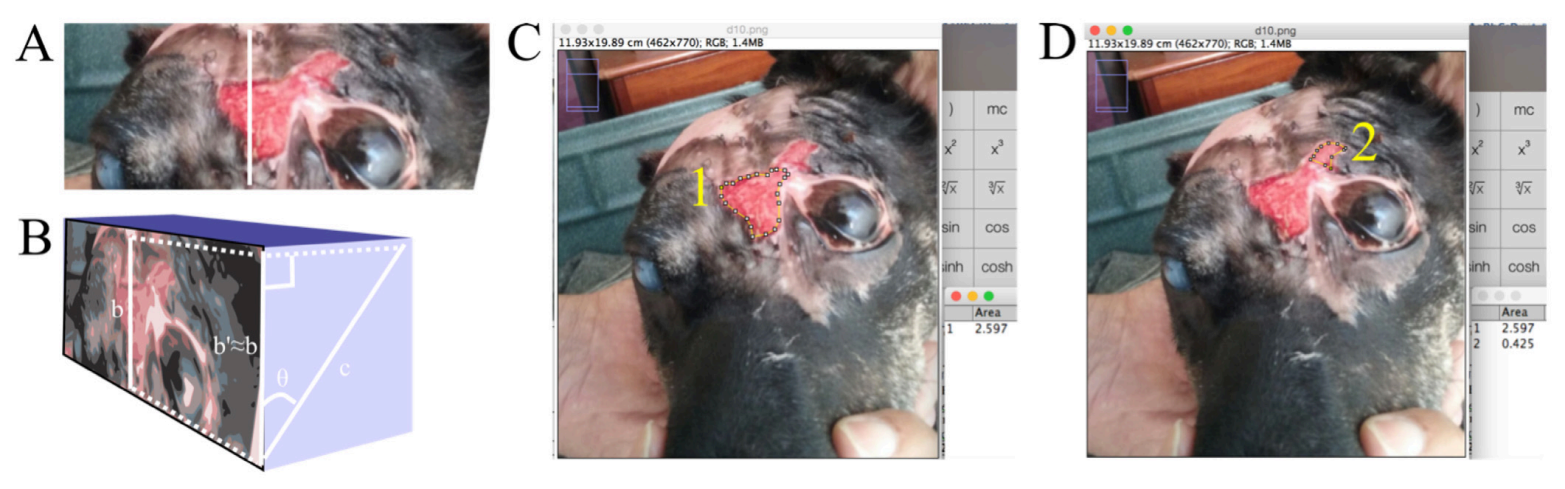
ImageJ software image analysis approach for quantitative size measurement of wounds - Part 2. ( **A**) The known distance (b) traced was, for photographs captured at an angle exactly normal to the wound plane, used for the “Set scale” function as-is, to trace the wound margin for use with the “Measure” function to obtain the wound area. ( **B**) For photographs captured at an inclined angle, a semi-quantitative estimate of the inclination angle (theta) was made and a simplified trigonometric system used to approximately determine the known distance's (c) adjacent cathetus b’ (b’≈b; the unknown distance in the camera plane) measurement in the *observer (camera) plane*. Then, using “Set scale”, the distance traced in b was taken as equal to b’=c*cos(θ). ( **C** and **D**) Thereafter, the wound margins were traced using the “Polygon selection” tool for subsequent measurement. This patient’s wound at this time point had formed a “pinch point” whereby the wound area above the eye had become discontinuous with the wound area adjacent to the eye (originally, these two were continuous) due to the advancing epithelialization, thus forming two distinct wound areas, 1 and 2, that were measured (using “Analyze-Measure”) as shown in **C** and **D**, respectively, and the sum of this *observer (camera) plane* wound area projected onto the true inclined *wound plane* using the trigonometric system explained above. The total wound area (whether a single area or the sum of distinct areas) was calculated and recorded.

Such analysis assumes the wound plane to be in the same plane as the observer (camera). Whenever photographs diverged from this standard photographing procedure, a semi-quantitative assessment was made to estimate the approximate *inclination angle* relative to the observer (camera). This angle was then used to estimate the true distance of wound plane by projection of the observer (camera) plane distance onto the inclined wound plane, using a simplified trigonometric approach, as outlined in Figure 4.

This method was applied to the photographs collected for every time point, for every individual, and every wound region, and a total wound area recorded in a table. The whole process was repeated, albeit using the same photograph, but redoing the set scale, the approximate polygon wound area/margin delineations, and measure function, thus producing a duplicate measurement for statistics. For each individual and time point, the mean wound area and the standard deviation were calculated, along with the coefficient of variation.

Another inferred parameter, the “Wound healing rate” was calculated using the mean wound areas, defined as:

$${\dot{A}}_{x}=\frac{\Delta A_{x}}{\Delta t_{x}}$$

where

$$\Delta A_{x}=A_{time\;point\;x}-A_{time\;point\;x-1}$$

is the current wound area (A _time point x_) measurement minus the previous wound area (A _time point x-1_) measurement, and

$$\Delta t_{x}=t_{x}-t_{x-1}$$

is the absolute time difference in days between the current wound measurement’s nominal time point and the previous’ nominal time point.

The calculated values for total wound area (A _time point x_) and wound healing rate (Ȧ _time point x_) were tabulated and plotted graphically against nominal time points. A third parameter, wound healing area at a given nominal time point, %A _time point x_, was defined as:

$$\%A_{time\;po\text{int}\;x}=\frac{\left(max\left(A\right)-A_{time\;po\text{int}\;\text{x}}\right)}{max\left(A\right)}\;$$

where

*max*( *A*)

is the maximum wound area for the series.

The choice of using the maximum value of the series, rather than the initial value, counteracted the effects of the imprecision of the method; at times, the second (D10) and third (D15) time point measurements, ended up having higher values than the initial value. Albeit not theoretically impossible (wound growing instead of shrinking/healing), the clinical, qualitative assessment did not suggest this was the case in any individual studied, and the choice was therefore made to use the maximum value as the common reference point for the series, as explained.

### Statistical analysis

The quantitative wound area measurements, using Image Analysis as the method, were obtained in duplicate. Basic statistical analysis was performed on these n=2 samples to provide a mean value (“AVERAGE function”), the standard deviation (“STDEV function”) and coefficient of variation (defined as the standard deviation divided by the mean value, for each sample). The software used for analysis was Excel 2011 (Microsoft Corporation, Redmond, WA, USA).

### Histology protocol

In the cases where pre-surgery and/or post-surgery biopsies of the affected area were collected, these were fixed in 10% formalin, embedded in paraffin, sectioned at 4 μm and subjected to the hematoxylin and eosin stain (H&E). The same protocol was applied to an unused biomembrane of identical specification to those used in the treatment group, to serve as a comparator for patient tissue histological samples. The stained tissue sections were visualized in an upright microscope with automatic scale bar incorporation.

## Results

### Patient characteristics

Patient N01 was approximately 6 years old (rescued animal, exact age unknown) at the time of surgery, female dog, with a skin lesion due to trauma (dog fight), disfigured fibrotic overgrowth of area adjacent to left eye with an area of approximately 10 cm ^2^.

Patient N04 was approximately 1 year old (rescued animal, exact age unknown) at the time of surgery, male cat, loss of skin equivalent to 2nd degree burn after car accident trauma with an area of approximately 120 cm ^2^ (note: approximately 40 cm ^2^ of the total lesion area was treated with the biomembranes, the remainder was treated using conventional wound treatment methods and healed at a slower rate than the biomembrane-treated areas).

### Qualitative wound healing: Photography and clinical assessment

**Figure 5.**
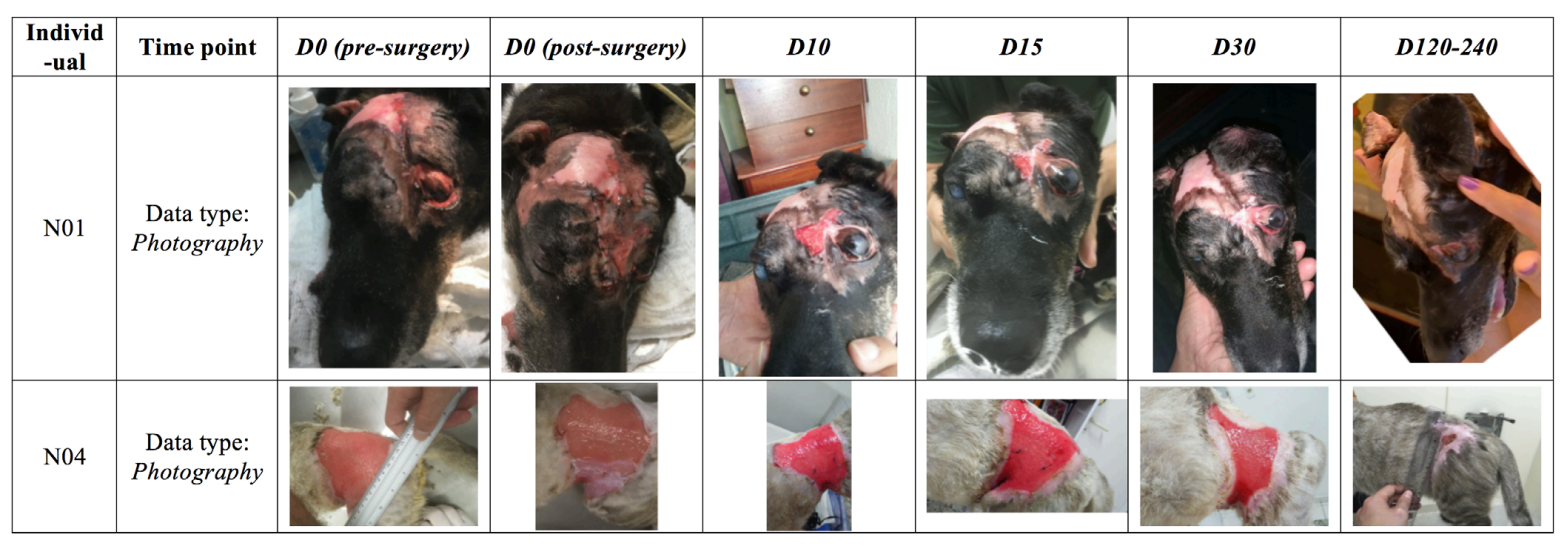
Photographs of areas treated with the biomembrane. In patient N01, old fibrotic scar tissue was removed surgically and two biomembranes applied and sutured to adjacent tissue. By time point D30, the wound had fully healed. In patient N04, only part of the lesion (approximately 1/3) was treated on D0 with the biomembrane (as seen in the D0 post-surgery photograph). The non biomembrane-treated area (without biomembrane in the D0 post-surgery photograph) had in fact been treated with another wound dressing two weeks before, but this treatment proved to be ineffective. The subsequent regeneration of the biomembrane-treated areas, opportunely led to a snowball effect that eventually would lead to the complete healing also of the non biomembrane-treated areas. The photograph in the last time point shown here, having a very wide bracket of Day 120 to 240, was in fact taken on day 129; follow-up visits were also done at the end of the D120-240 time point interval, but photographs are not available. Nonetheless, the clinical data obtained at this final assessment confirm complete wound closure with clinically excellent parameters, such as suppleness, robustness, lack of contracture and with unrestricted mobility in the region in question (as seen on examination of the patient). The photographs provided basis for these *qualitative* assessments, in addition to the *quantitative* assessment aided by image analysis. In the case of N04, only the areas treated with the biomembrane *de facto* were used in the statistical analyses (even though the snowball effect led to healing of non biomembrane-treated areas, but at a slower rate).

The photography series presented a favorable outcome in terms of wound closure with the formation of healthy skin in the areas treated, in both patients studied in this interim report. In both patients, vascularization of the biomembrane-treated areas was observed clinically by the marked redness of the areas treated. The *neo-*skin that was formed presented itself as supple and robust, as opposed to a tissue that could form itself as hard and/or fragile, respectively. Moreover, in both patients, the areas treated ended up, at the end of the follow-up period, having a partial or complete coverage of fur, indicating the possible formation of new hair follicles in the treated areas, or, alternatively, a migratory mechanism whereby hair follicles in adjacent healthy areas were tugged toward the center of the wound/treated area, as part of the gradual wound closure process. The photographs collected are shown in Figure 5. 

Worthy of an additional explanation, N04’s fur coat color/tonality changes significantly from D30 to the last time point, D120–240. Such changes are not uncommon in cats, especially in individuals that have not yet reached adult age, as was the case here. Other variables, such as temperature/season change (here, fall-winter), sunlight exposure, diet, hormones and stress also play a role, as do genetics and illness (here, the significant change may be due to the transition from a “sick” or “recovering” phenotype to a “healthy” phenotype).

### Quantitative wound healing: Image analysis

**Figure 6.**
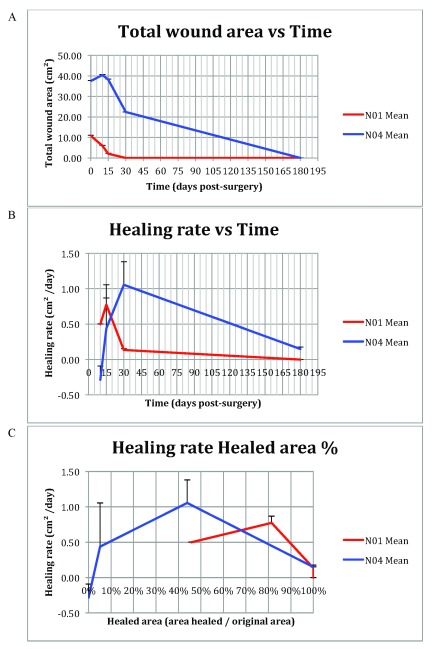
Graphs depicting data from Table 2. ( **A**) Total wound area vs Time. ( **B**) Wound healing rate vs Time. ( **C**) Wound healing rate vs Wound healing percentage. Error bars indicate +1 SD from mean data point.

The photographs collected were analyzed as described in the *Methods*, giving rise to values for wound area, healed area and healing rate, as tabulated in Table 2. Graphs for *Wound area* vs *Time*, *Healing rate* vs *Time* and *Healing rate* vs *Healed area*, based on the values shown in Table 2, are shown in Figure 6. The wounds of the two patients studied differed somewhat in size, which in turn made it more difficult to generalize features of healing in the wounds and individuals treated. However, it was noted that, in general, the *healing rate* accelerates during the first phase of the wound healing, reaching a maximum at D15–30. When looking at the *Healed area percentage* at maximum *Healing rate*, the results indicate that maximum *Healing rate* coincides with a *Healed area percentage* of around 50% or more. Upon investigating the general shape of the curves, in particular in Figure 6B curve depicting *Healing rate* vs *Time*, the first part of the curves (from the beginning of the data set to maximum value) have a comparable, accelerating shape, but the latter part (from maximum value to the last data point) have differing shapes, possibly owing to lack of suitable complementary time/data points between D30 and D120–240.

**Table 2.** Quantitative wound healing parameters. Obtained from analysis of data collected at the time points as per the study protocol, from Day 0 to Day 120–240. The values for parameter A, wound area, were calculated using an image analysis approach of photographs taken at the different time points, from which the two other parameters: %A, healed area; and Ȧ, healing rate, were calculated. *x*, mean; *SD*, standard deviation; *CV*, coefficient of variation.

				Time point				
Patient	Parameter	Statistical descriptor	Units	D0	D10	D15	D30	D120–240
*N01*	*A* *wound area*	*x̄*	*cm ^2^*	10.87	5.90	2.02	0.00	0.00
		*SD*	*cm ^2^*	0.16	0.21	0.25	0.00	0.00
		*CV*	*-*	1.5%	3.6%	12.4%	n/a	n/a
	*%A* *healed area*	*x̄*	*-*	0%	46%	81%	100%	100%
		*SD*	*-*	0.0%	1.1%	2.6%	0.0%	0.0%
		*CV*	*-*	0.0%	2.4%	3.2%	0.0%	0.0%
	*Ȧ* *healing rate*	*x̄*	*cm ^2^/day*	n/a	0.50	0.78	0.13	0.00
		*SD*	*cm ^2^/day*	n/a	0.01	0.09	0.02	0.00
		*CV*	*-*	n/a	2.0%	11.5%	15.4%	0.0%
*N04*	*A* *wound area*	*x̄*	*cm ^2^*	37.54	40.40	38.22	22.37	0.00
		*SD*	*cm ^2^*	5.74	3.79	0.69	4.20	n/a
		*CV*	*-*	15.3%	9.4%	1.8%	18.8%	0.0%
	*%A* *healed area*	*x̄*	*-*	7.3%	0.0%	5.1%	43.9%	100.0%
		*SD*	*-*	5.5%	0.0%	7.2%	15.6%	0.0%
		*CV*	*-*	75.3%	n/a	141.2%	35.5%	0%
	*Ȧ* *healing rate*	*x̄*	*cm ^2^/day*	n/a	-0.29	0.44	1.06	0.15
		*SD*	*cm ^2^/day*	n/a	0.20	0.62	0.33	0.03
		*CV*	*-*	n/a	-69.0%	140.9%	31.1%	20.0%

### Histology

As described in the *Methods*, pre-surgery histology would only be done with the patient’s pre-existing scar tissue, removed during the first stage prior to the application of biomembranes. Moreover, post-surgery histology of a biopsy taken from the biomembrane-treated healed wounds would only be done in conjunction with another surgical procedure executed at a later date, i.e., no biopsy would be taken solely for the purpose of obtaining post-surgery histology data.

**Figure 7.**
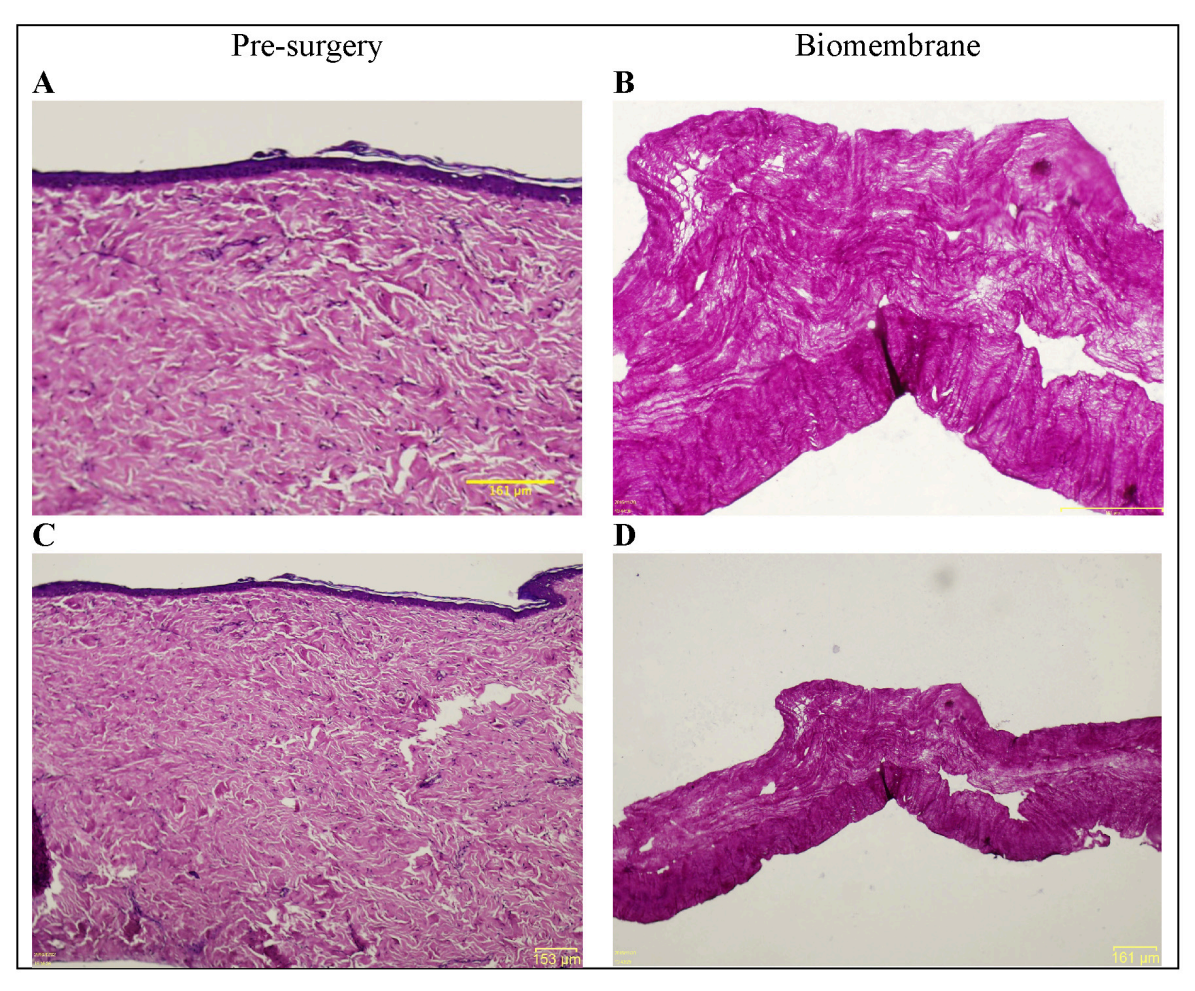
Hematoxylin and eosin (H&E) histomicrographs. ( **A** and **C**; 20 × and 4 × magnification of same specimen) Hematoxylin and eosin (H&E) histomicrograph of N01’s pre-surgery tissue in the treatment area, exhibiting numerous hallmarks of fibrotic scar tissue. Epidermis is flattened, with no undulation on the surface, no epidermal rete ridges extending into the dermis, and no dermal papillae. The uppermost papillary dermis presents a dense collagenous matrix, and the lowermost, reticular dermis exhibits highly acidophilic regions with denser bundles of collagen. ( **B** and **D**; 20 × and 4 × magnification of same specimen) H&E histomicrograph of a representative collagen biomembrane (i.e., equivalent to the implanted biomembranes prior to their utilization). Horizontally aligned thin collagen fibers with some larger pores interspersed throughout the structure, along with smaller pores with a more uniform distribution. The lowermost part of the structure presents increased acidophilicity and collagen density. This particular part of the histomicrograph shows the local elevation of approximately 100 μm in height. These elevations are part of the characteristics of the biomembrane, and are present throughout the structure, spaced approximately 2.5 mm from one another.

In this interim report, patient N01’s pre-existing scar tissue was removed, and the corresponding histomicrograph is shown in Figure 7 (A and C). In the case of patient N04, no pre-existing scar tissue was available for histology and the biomembranes were applied directly to the wound bed as described above. At the time of writing, no later surgical procedures were scheduled for either of the two individuals covered by this interim report, and, as such, no post-surgery histology data is available. Figure 7 (B and D) shows a histomicrograph of a biomembrane representative of the biomembranes applied in the study.

Dataset 1. Biomembrane data files. This dataset contains the unedited photographs from the study http://dx.doi.org/10.5256/f1000research.15138.d206373
Copyright: © 2018 Kaasi A et al. 2018 Data associated with the article are available under the terms of the Creative Commons Zero "No rights reserved" data waiver (CC0 1.0 Public domain dedication).

## Discussion

The present clinical trial did not have a randomized sample assigned to a treatment and a placebo arm; rather, the sole sample group was the treatment group, and a quasi-experimental control group (pseudo-placebo arm; Figure 1) was taken as the clinicians’ assessment of how the individual in question would fare *without* the treatment with the biomembrane. In both patients reported in this interim report, the biomembranes were capable of stimulating formation of quality skin tissue and complete wound closure in the time frame studied, indicating efficacy. The clinicians’ assessment (J.F.L.N. and M.H.S.C) was that the wounds would take approximately 3 × longer to heal compared to the time it took using the biomembrane treatment, or, possibly, the wounds would not have healed at all, or formed fibrotic scar tissue, indicating superiority compared to conventional wound therapy. No adverse effects were noted, indicating safety. In addition to the objectives of the study pertaining to skin repair, an additional apparent benefit of the biomembrane treatment was seen with the apparent stimulation of fur growth, a characteristic that will be investigated in more detail for subsequent patients enrolled in the study. The image analysis method for quantitative size assessments is robust and accurate for photographs taken perpendicular to the wound (commercial systems also exclusively mandate perpendicular image capturing), but when taken at an angle, even with the trigonometric correction as detailed herein, the final size will suffer and not present good accuracy. Ideally, all photographs should be taken completely perpendicularly to the wound to maximize accuracy (and precision).

The enrolment of patients has been slow, due to cost considerations, but agreements have been made with new partner clinics to ensure the total number of 15 patients is reached by the end of the study period. We believe the biomembrane is well suited for wound healing in veterinary patients, and foresee other uses as well, e.g. as a periosteum substitute. Moreover, due to the similarities between the veterinary medical device studied in this report and human medical devices based on tissue engineering technologies, we expect that the study may have repercussions beyond the veterinary domain and into the human domain, and vice versa.

## Data availability

The data referenced by this article are under copyright with the following copyright statement: Copyright: © 2018 Kaasi A et al.

Data associated with the article are available under the terms of the Creative Commons Zero "No rights reserved" data waiver (CC0 1.0 Public domain dedication). 

Dataset 1: Biomembrane data files. This dataset contains the unedited photographs from the study. DOI, 10.5256/f1000research.15138.d206373 ( Kaasi *et al*., 2018).

## Footnotes

1 There are as yet no widely known veterinary clinical trial registries, apart from the American Veterinary Medicine Association’s (AVMA) Animal Health Studies Database. This database is the first of its kind, and was only 4 months old at the time the present trial was conceptualized and commenced (October 2016). The authors only became aware of its existence in subsequent months, and the study details were submitted to the database thereafter; however, the AVMA informed the authors that they were unable to accept studies from outside the U.S.A. and Canada, with the reason being they do not have the resources to consider study listings in the context of animal use laws and regulations that vary from country to country. As such, it was decided to include the study protocol as a Supplementary File to this article. It is also available on the Eva Scientific website ( http://www.evascientific.com/products/a161012001/).

## Supplementary material

Supplementary File 1: CONSORT checklist.

Supplementary File 2: Study protocol.

Supplementary File 3: Footage of application of double-layer regenerative collagen biomembrane.
