# Supplementary material for: Regenerative collagen biomembrane: Interim results of a Phase I veterinary clinical trial for skin repair
Source: F1000Res. 2018 Jun 12;7:729. [Version 1] doi: 10.12688/f1000research.15138.1 (PMC6338246; doi:10.12688/f1000research.15138.1)
Supplement: Supplementary file 3 [file f1000research-7-16492-s0002.tgz › 1d8c1ac5-2a35-42fc-beeb-c3bf917f7f70.pdf]

Clinical trial making use of acellular collagen biomembrane. Currently recruiting patients.

Animal Health Clinical Trials Database - Maintained by Eva Scientific

For additional information or instructions on how to enroll in the clinical trial, please contact the study contact as per the below study protocol.

Published: 30 May 2018
Last updated: 07 June 2018

1 - Clinical Trial details:
Scientific title: Regenerative collagen biomembrane: A Phase I veterinary clinical trial for skin repair
Public title: Regenerative collagen biomembrane for skin repair
Reference number: A161012001

2 - Clinical Trial contact:
Name: Andreas Kaasi
E-mail: ak@evascientific.com
Country: Brazil
Telephone: +551141141291
URL: http://www.evascientific.com/products/a161012001/

3 - Principal Investigator contact:
Name: Andreas Kaasi
E-mail: ak@evascientific.com
Country: Brazil
Telephone: +5511983076000
URL: http://www.evascientific.com/products/a161012001/
Affiliation: Eva Scientific Ltd., São Paulo, Brazil; Biofabris- National Institute of Biofabrication, Campinas, Brazil; Unicamp-University of Campinas, Brazil

4 - Clinical Trial summary
BACKGROUND
The availability of commercial tissue engineered skin repair products for veterinary use is scarce or non-existent. To assess features of novel veterinary tissue engineered medical devices, it is therefore reasonable to compare with human devices. In addition, during the development and regulatory approval phases, these human medical devices, identified as comparable to the novel veterinary device, may serve as predicate devices and accelerate approval in the veterinary domain. As part of this study, we are comparing the features and underlying technology behind five such possible predicate devices, approved for human use, employing similar tissue engineering technologies as the present regenerative collagen biomembrane, which was the object of investigation for the present Phase 1 veterinary clinical trial. The purpose of the study was to evaluate safety and efficacy of the biomembrane for use in skin repair indications.

METHODS
Study design: 15 patients, dogs and cats, male/female, 2 cm, wound depth equivalent to 2nd/3rd degree burns, studied from Day 0 to Day 120-240 post-application of the biomembrane. Surgical regimen: Wound beds were prepared and acellular collagen biomembranes (Eva Scientific, São Paulo, Brazil) applied directly onto the wounds, and sutured at the margins to the patient's adjacent tissue.
Wound assessment techniques: Qualitative (appearance and palpation) and quantitative (based on Image Analysis of photographs).
Outcomes: Wound size over time, healing rate, general skin quality and suppleness.

Diagnosis: Wound
Intervention type: device
Intervention name: Acellular collagen biomembrane
Intervention control group: No control group
Primary outcome event: Wound closure; healing rate; macroscopic quality of skin / hardness; microscopic quality of skin
Primary outcome method of measurement: Photography and clinical assessment; image analysis; photography, clinical assessment and palpation; histology
Primary outcome endpoint: If complete wound closure is achieved by the last, long-term follow-up time point (Day 120-240): End point for that animal's treatment = this follow-up event.
If wound closure incomplete by the end of the period (D240): End point ≈ D240.
Secondary Outcome #1 Event:
Secondary Outcome #1 Method of measurement:
Secondary Outcome #1 End point:
Secondary Outcome #2 Event:
Secondary Outcome #2 Method of measurement:
Secondary Outcome #2 End point:

5 - Clinical Trial classifications:
Clinical Trial type: Interventional
Patient randomization: No

Investigator 'Masked': No
Owner 'Masked': No
Species: Canine, feline
Primary field of medicine: Soft tissue
Subcategory field of medicine: Integumentary surgery (incl. skin flaps/grafts)

6 - Enrollment considerations:
Inclusion criteria: Species: dog or cat; Sex: male or female; Age 2 cm (L/W, diameter as per geometry)
Exclusion criteria: Epidermis-only lesions (equivalent to 1st degree burn); Skin lesions stemming from chronic disease
Benefits: Healing of skin lesions considered difficult to heal
Risks: Equal or sub-par healing compared to traditional wound healing protocols
Funding source: Federal agency, Company, Institution
Funding names: Government agency: CNPq and FAPESP; Corporate: Eva Scientific Ltd.; Academic institution: Biofabris-National Institute for Biofabrication
Recruitment status: Recruiting
Start date: 12 Oct 2016
End date: 18 Oct 2019
Financial incentives: Partially funded (Owner cost expected < US$500)
Primary location: Sorocaba Veterinary Hospital, Sorocaba, Brazil
Secondary location(s):

7 - Clinical trial search terms, compliance and results:
Keywords: collagen biomembrane, tissue engineering, wound healing
Study compliance: Yes
Study results:
