# Supplementary material for: Regenerative collagen biomembrane: Interim results of a Phase I veterinary clinical trial for skin repair
Source: F1000Res. 2018 Jun 12;7:729. [Version 1] doi: 10.12688/f1000research.15138.1 (PMC6338246; doi:10.12688/f1000research.15138.1)
Supplement: Peer review history

# Peer review: Referee response for version 1

**DOI:** 10.5256/f1000research.16492.r41549

**Published:** 2019-01-11

**Authors:** Kalan Bastos Violin

**Affiliations:** 1 Material Science and Technology Center, Nuclear and Energy Research Institute (IPEN) , São Paulo, Brazil

The article seems to provide enough evidence for the proposed goal, which basically was to induce skin repair in a critical size wound equivalent to 2nd to 3rd degree burns in depth, using an engineered biomembrane based on collagen type I.

 The biggest challenge was to gather enough subjects eligible to the study, presenting only 2 cases isn't ideal, but expected since real cases for clinical trials are difficult to respond to all criteria to be included. For further studies these criteria should be flexed to maximize the number of subjects included, specially if the exclusion of those were related to unsuccessful results, cut to not compromise the product. A larger number of subjects is desirable and for clinical trials mandatory. Nevertheless for this level of initial trial and concept it is acceptable and the results were remarkable.

 Another point to be kept for the following research is to give a molecular approach to the biopsies. Basic histology just present the morphological aspects of the tissue and it would be interesting to gather from these same samples an immunohistochemistry analysis to characterize the molecular content of the formed tissue looking for proteins related to repair and healing, but also for inflammatory and regulatory markers that play an important role in those process. This would give a enormous depth to the consolidation of the material and present more reliable and straightforward data for comparison.

I have read this submission. I believe that I have an appropriate level of expertise to confirm that it is of an acceptable scientific standard.

# Peer review: Referee response for version 1

**DOI:** 10.5256/f1000research.16492.r34932

**Published:** 2018-09-27

**Authors:** Iris Ribitsch

**Affiliations:** 1 Department for Companion Animals and Horses, Veterm, University Equine Hospital,Vetmeduni Vienna, Vienna, Austria

Introduction:

 The introduction focuses on an important topic which is drawing more and more attention in medical sciences: The idea of “one health” - the collaborative effort of multiple disciplines – to attain optimal health for people, animals and the environment. The authors point out the necessity to conduct veterinary clinical trials in a standardized manner in order to obtain valid and meaningful results to facilitate the translation into human medicine. Currently there is a lack of global consensus concerning the rules and regulations governing veterinary clinical trials.  

 However, this discussion (although very important) should not make the bigger part of the introduction. Instead please include a discussion of solid scientific evidence on the similarities and discrepancies of human, feline and canine wound healing and why it may be valid to extrapolate results from one species to the other. Furthermore, please offer and overview of the research question, the state of the art in the field, how the current state may be improved, what is new about the collagen membrane used in this study compared to other similar products and how and why the collagen membrane employed in this study may therefore contribute to an improvement of the current state of the art. Currently the collagen membrane, which is the core of this paper, is not described whatsoever.

 Methods:

 The materials and methods section offers a description of the study design of the envisaged phase 1 clinical trial. It gives in detail guidelines how to proceed at certain stages in the course of the study and based on findings which may be encountered within the scope of the study (e.g. “If the affected area had been subject to natural scarring, with the formation of hard, unhealthy, fibrotic scar tissue, this was surgically removed, exposing subcutaneous tissue. In this case, the tissue would be fixed in 10% formalin and submitted to histological analysis, using standard procedures. If the treatment area was an unhealed, deepithelialized wound bed, but with no fibrotic scar tissue formation, then shallow scalpel incisions were made scattered throughout the treatment area, causing local bleeding, with the aim of promoting regeneration and integration with the later application of the collagen biomembranes.”

 “If any adverse effects were confirmed, in the first 1–2 days, then the biomembranes were excised or scraped off host tissue. If adverse effects were confirmed in later stages (>D3), the biomembranes were not excised due to the fact that by this time they had absorbed significantly into the host tissue, rendering removal difficult or impossible. In this case, the patients were treated symptomatically and added to the “Discontinued intervention” group…”).

 Also detail on how data should be obtained and by whom are included (“When possible, a qualitative assessment of skin tissue quality would be done, either by a researcher associated with the study or by the owner. If another surgical procedure was scheduled for a study participant, for any reason, a small skin biopsy was attempted to be taken from the treatment area….”).

 However, the materials and methods should provide detailed information on methods and analysis carried out particularly for the current 2 patients included into the study.

 The anaesthesia regimen which is described in great detail is not so relevant for the interpretation of the study results as e.g. the preparation of the skin prior to surgery which may have considerable influence on the healing process. Please provide more information.

 Results:

 Following up clinical patients especially long term is very challenging and depends on the cooperation of the animal owners and their willingness and time to take their animals to the follow up examinations etc. Using series of pictures which can be taken either by the owners or the treating veterinarian at defined time points to document the healing process will certainly facilitate cooperation. Detailed instructions when and how (distance camera to animal, angle camera to wound etc.) are offered in the methods section. However, the photos in the paper (Figure 5) do unfortunately not look standardised even when comparing only the pictures taken from one patient, which hampers comparability.

- Is the work clearly and accurately presented and does it cite the current literature? No.
- Is the study design appropriate and is the work technically sound? No.
- Are sufficient details of methods and analysis provided to allow replication by others? Yes
- If applicable, is the statistical analysis and its interpretation appropriate?

         Meaningful statistical analysis on n=2 is impossible.

          A detailed description of the results is more useful in this case.

- Are all the source data underlying the results available to ensure full reproducibility? Yes
- Are the conclusions drawn adequately supported by the results? Partly

 

 The results of the study are interesting and encouraging and (so I am convinced) worth further research efforts.

 However, in order to improve the quality of the clinical study standardisation should be increased, the photo documentation should be improved, mandatory re-checks with the treating veterinarian should be implemented during which a healing score is obtained based on healing rate and a qualitative assessment of newly formed skin tissue. Histologic evaluation pre and post-surgery should be implemented. Also inclusion of a control group would considerably increase the quality of the study. The clinicians assessment (“The clinicians’ assessment was that the wounds would take approximately 3 × longer to heal compared to the time it took using the biomembrane treatment, or, possibly, the wounds would not have healed at all, or formed fibrotic scar tissue, indicating superiority compared to conventional wound therapy.) and a ‘quasi-experimental control group’ are not sufficient. I certainly agree with the author´s that it wouldn´t be possible and ethically debateable to include a control group receiving no treatment, but a control group receiving the current gold standard of wound treatment would be feasible and still increase the quality of the study.

 I strongly suggest publishing the results from the two patients as case reports because they are valuable and interesting for the scientific community and veterinary clinicians, but at the current stage unfortunately not sufficient for an original research article. However, I am looking forward to read more and see more results to come from further patients enrolled into this interesting and promising clinical trial and whish the authors all the best for the continuation of their study.

I have read this submission. I believe that I have an appropriate level of expertise to confirm that it is of an acceptable scientific standard, however I have significant reservations, as outlined above.

# Peer review: Referee response for version 1

**DOI:** 10.5256/f1000research.16492.r35668

**Published:** 2018-07-19

**Authors:** Adam Bohr, Kaveh Memarzadeh

**Affiliations:** 1 Barts and The London School of Medicine and Dentistry, Queen Mary University, London, UK; 2 Orthopaedic Research UK, London, UK; 3 Mechanical Engineering Department, University College London, London, UK; 4 Department of Pharmacy, Faculty of Health and Medical Sciences, University of Copenhagen , Copenhagen, Denmark

The manuscript entitled, “Regenerative collagen biomembrane: Interim results of Phase I veterinary clinical trial for skin repair” by Kaasi et al explores the application of acellular collagen membranes for veterinary wound healing purposes in dogs and cats. The study provides the preliminary results to what may be considered a phase I clinical study on animal subjects that had been victims of injuries with the immediate need of care. The manuscript is well written with a good structure and the data presented is interesting and worth publishing. There are, however, some minor clarifications and modifications that are suggested to improve the manuscript before publication. 

 1: The study presents an interesting idea of letting veterinary clinical trials pave the way for human use and equivalent therapy. Currently, there is a lack of global consensus concerning the rules and regulations governing veterinary clinical trials. The establishment of such rules and regulations could possibly make the translational process from veterinary subjects to man more flexible for some medical devices by providing substantial data and thereby allow a faster route to regulatory approval. It is suggested that the authors add a couple of sentences in the discussion section on the potential prospect of the proposed wound healing product for human applications and the long-term objectives or plans of the authors for the clinical translation of the product.

 2. There is not sufficient information on the characteristics of the collagen biomembrane to fully differentiate and appreciate the current approach. Please provide more information concerning the main attributes of the collagen biomembrane and a comparison with similar marketed products or products in development. How does the presented collagen membrane differ from other biomembranes including cell-incorporating and synthetic alternatives?

 3. It is somewhat unclear with the current status of the animal studies. Please provide additional information regarding the current status with the number of completed animal subjects and information and timeline for the remaining studies and subjects

 4. If there were any adverse effects noticed such as infection and necrosis after application of the biomembrane, what measures are there to investigate this further? Did this occur in this current study? If yes, please provide some insight as to why you think this was the case.

 5. Regarding photographing the patients, how do the authors explain the method of ‘Semi-quantitative assessment’? More detail is required about this method. Going forward, the method of photographing animals should be standardised in order to lessen the chance of errors in the study. 

 6. There are no clear controls for each model presented. We appreciate that this is a very challenging task to achieve at this stage, however going forward a ‘quasi-experimental control group’ will not suffice. We would advise the researcher to have solid controls in place when recruiting subjects for the next studies.  

 7. The histological evidence provided here is very limited. This needs to be improved upon significantly. The histological data presented in this paper is only for one patient and only in a pre-surgical stage ( A and C). The researchers need to elaborate on the reasons why they were unable to provide small histological samples from patients after treatment. The local elevations present on the biomembrane and pores are highlighted in Figure 7, however, the biological significance of each characteristic is missing. It would also be great if the researchers made the scales more visible on the H&E images.

 8- The authors refer to an apparent fur growth on one of the patients, however, this is not backed by supporting evidence as the numbers are very low. Please refrain from using terms such as ‘benefit’ and ‘stimulation’ in these instances and just state what has been observed and report fully when enough evidence is available. 

 9. Please provide an indication of the time generally required to get a veterinary medical device on the market.

We have read this submission. We believe that we have an appropriate level of expertise to confirm that it is of an acceptable scientific standard.
